# Evaluating a Serious Gaming Electronic Medication Administration Record System Among Nursing Students: Protocol for a Pragmatic Randomized Controlled Trial

**DOI:** 10.2196/resprot.9601

**Published:** 2018-05-28

**Authors:** Richard Booth, Barbara Sinclair, Josephine McMurray, Gillian Strudwick, Gavan Watson, Hanif Ladak, Merrick Zwarenstein, Susan McBride, Ryan Chan, Laura Brennan

**Affiliations:** ^1^ Arthur Labatt Family School of Nursing Faculty of Health Sciences Western University London, ON Canada; ^2^ Business Technology Management/Health Studies Wilfrid Laurier University Waterloo, ON Canada; ^3^ Information Management Group Centre for Addiction and Mental Health Toronto, ON Canada; ^4^ Teaching Support Centre Western University London, ON Canada; ^5^ Department of Medical Biophysics Faculty of Engineering Western University London, ON Canada; ^6^ Centre for Studies in Family Medicine Schulich School of Medicine and Dentistry Western University London, ON Canada; ^7^ School of Nursing Texas Tech University Health Sciences Center Lubbock, TX United States

**Keywords:** video games, high fidelity simulation training, nursing education, medication errors

## Abstract

**Background:**

Although electronic medication administration record systems have been implemented in settings where nurses work, nursing students commonly lack robust learning opportunities to practice the skills and workflow of digitalized medication administration during their formative education. As a result, nursing students’ performance in administering medication facilitated by technology is often poor. Serious gaming has been recommended as a possible intervention to improve nursing students’ performance with electronic medication administration in nursing education.

**Objective:**

The objectives of this study are to examine whether the use of a gamified electronic medication administration simulator (1) improves nursing students’ attention to medication administration safety within simulated practice, (2) increases student self-efficacy and knowledge of the medication administration process, and (3) improves motivational and cognitive processing attributes related to student learning in a technology-enabled environment.

**Methods:**

This study comprised the development of a gamified electronic medication administration record simulator and its evaluation in 2 phases. Phase 1 consists of a prospective, pragmatic randomized controlled trial with second-year baccalaureate nursing students at a Canadian university. Phase 2 consists of qualitative focus group interviews with a cross-section of nursing student participants.

**Results:**

The gamified medication administration simulator has been developed, and data collection is currently under way.

**Conclusions:**

If the gamified electronic medication administration simulator is found to be effective, it could be used to support other health professional simulated education and scaled more widely in nursing education programs.

**Trial Registration:**

ClinicalTrials.gov NCT03219151; https://clinicaltrials.gov/show/NCT03219151 (Archived by WebCite at http://www.webcitation.org/6yjBROoDt)

**Registered Report Identifier:**

RR1-10.2196/9601

## Introduction

### Background

Ensuring clinician sciences students have access to appropriate and pedagogically robust clinical learning opportunities is important to generate future cohorts of competent practitioners. The wide-scale adoption of high-fidelity patient simulation tools into clinical education (ie, nursing, medicine, and pharmacy) [1,2] is increasingly being extended and amplified through the use of technology-enabled (ie, online) serious gaming and other gamified approaches [3,4]. Serious gaming or gamification has been defined by Koch et al [5] as “the use of game design elements in nongame contexts” for the purposes of engaging learners in solving complex problems. In nursing education, there is currently minimal use of immersive, technology-enabled, or gamified approaches for students to practice clinical skills and judgment [6,7]. Commonly, in-person, simulated practice with high-fidelity patient simulation provides students the only functional opportunity to learn various clinical skills (eg, medication administration) before interacting with real patients in health care environments such as hospitals and in the community [7]. Although this type of in-person simulation has been found to be extremely effective toward generating clinical competency and skills [8,9], virtually all this kind of learning is contingent on the availability of, and access to, a physical simulation laboratory. Given the increasing enrollment sizes and current financial restraints present in many universities and colleges, scalable, cost-effective, and authentic [10] technology-enabled approaches (including gamification) for clinical nursing education are warranted.

### Electronic Medication Administration and Nursing Education

Electronic medication administration record (eMAR) systems have become more popular in many health care environments over the last decade, as a mechanism to improve medication safety. In brief, an eMAR system is a type of clinical technology that a health care provider (ie, nurse) uses to both validate and record the administration of medications. eMAR systems commonly use barcode technology to ensure that the correct medication is being administered to the correct patient at the correct time, dosage, and route [11]. Nurses are currently one of the largest user groups of eMAR technology [12,13]; therefore, it is essential that eMAR medication administration skills-building is fully embedded within nursing education. In clinical practice settings where eMAR systems are used effectively, statistically significant reductions in medication errors in comparison with those using paper-based medication administration processes have been reported [14]. Due to ongoing high rates of adverse medication events in both Canada and the United States [15,16], clinical technology such as eMARs may be essential for the reduction of medication-related errors [17,18].

One significant issue within nursing clinical education is competency and self-efficacy development in safe medication administration practices. Commonly, nursing students receive theoretical courses related to pharmacology and also clinically focused courses during the early years of their undergraduate education. Before students administering medications to real patients, learners usually participate in simulated, in-person learning opportunities within a laboratory setting. In these controlled learning environments, students can practice skills and further integrate their theoretical knowledge of pharmacology with professional practice skills related to medication administration. With recent advances in clinical technology such as eMAR systems [19], the complexity of the medication administration process has become a recognized nursing education and simulation issue [11,20-22]. Past research examining eMAR in nursing education simulation has outlined a range of factors limiting student success in this new modality of medication administration—namely, the amount of time and access students have to learn the unique workflows and clinical decision-making requirements in a digitized medication administration process [22]. There has been little research into whether serious gaming or gamification might be used as a tool to educate students in the complex tasks and processes inherent in medication administration [3,23], outside of the simulation laboratory. Finally, there are no known studies that examine whether medication administration gaming, in advance of simulated clinical practice, results in better nursing student learning, self-efficacy, and performance outcomes.

### Objectives of the Study

The main objectives of this study are to examine whether the use of an eMAR simulation serious game (1) improves nursing students’ attention to medication administration safety within simulated practice, (2) increases student self-efficacy and knowledge of the medication administration process, and (3) improves motivational (ie, attention, relevance, confidence, and satisfaction) and cognitive processing attributes (ie, invested mental effort and difficulty to learn game) related to student learning in a technology-enabled environment. Three specific research questions will be addressed in this study:

1. Does the use of an eMAR simulation game improve the eMAR medication administration safety for nursing students during in-person, simulated clinical practice?
2. Does the use of an eMAR simulation game improve learner self-efficacy and knowledge toward eMAR medication administration processes in nursing students for in-person, simulated clinical practice?
3. Does the use of an eMAR simulation game improve the motivational and cognitive processing attributes of nursing students regarding their learning of eMAR medication administration in a technology-enabled environment?

## Methods

### Development of a Prototype Electronic Medication Administration Record Simulation Game

At a large Canadian university in southern Ontario, a simulated eMAR system named the *SMART eMAR* was designed, developed, and implemented by faculty and students [24,25]. The SMART eMAR is used within the university’s clinical simulation laboratory (CSL) and provides students the opportunity to practice medication administration processes during various in-person, simulated clinical practicum courses. The SMART eMAR is a cost-effective, scalable, and customizable platform that resembles a real clinical eMAR system in terms of both interface and usability. The system contains all the major decision support elements and functionalities of a real eMAR system, including color-coded decision support related to incorrect barcode scans of medication or patient identification bracelets, barcode scanning capabilities of a host of inert medications used in the CSL, auto-populating data fields for time stamps and date, and qualitative text fields for nurse signatures or commenting. Both the hardware and software components of the SMART eMAR are mounted onto hospital-grade Ergotron mobile carts to replicate the usability and physical appearance of a clinical workstation on wheels.

First, although the SMART eMAR was designed to have a simplistic eMAR user interface (vs eMAR platforms found in acute care hospital environments), students had a difficult time learning how to effectively use and administer medications using this system [11]. Findings from this previous research also suggested that nursing students did not feel that they possessed enough knowledge regarding the process of electronic medication administration or the clinical judgment factors (ie, specific time-dose dependencies associated with some medications) to effectively use the SMART eMAR in simulated practice [11,20]. Second, students reported that they did not feel they had adequate time with the SMART eMAR to fully understand the work processes required to use the system confidently [11]. These findings are consistent with other research examining medication administration by nursing students [26]. Gamification of the eMAR process was selected by the research team to be both an innovative and logical solution to address the learning barriers currently faced by students [11].

**Figure 1:**
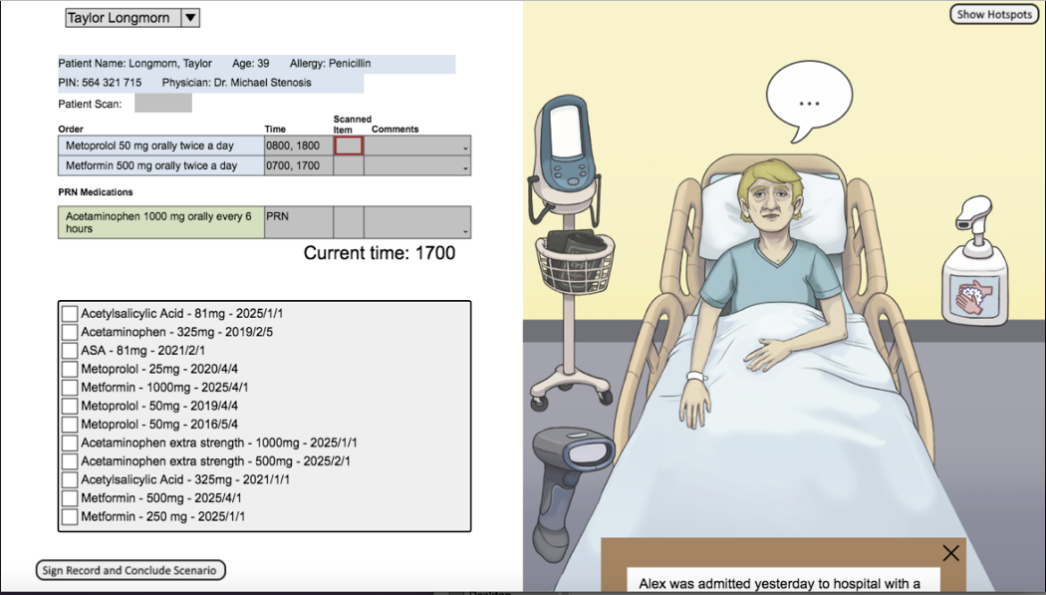
Screenshot of the electronic medication administration record (eMAR) simulation game’s user interface and various player-manipulable objects.

To assist with game development of an eMAR simulation game, the research team partnered with a video game company (Mikutech, Joydrop Ltd, London, Canada) that has significant experience in the areas of video game and software development, including the specialized fields of education, engineering, and health care [27]. Drawing on previous research and medication administration workflow on the SMART eMAR [11,24], 2 main teaching-learning directions were targeted in the eMAR simulation game development. First, the main objective of the eMAR simulation game was to afford students a virtual and immersive opportunity to practice medication administration using an eMAR system in a structured environment that provides feedback consistent with previously published best practices [11,20,25]. Second, the simulation game was planned to gamify the medication administration process for a range of patient’s clinical presentations, with multiple feedback loops for each decision point along the medication administration pathway. Using a finite-state machine logic, each of these feedback loops could result in either *success, qualified success,* or *failure* in the administration process, which would be reported back to the student upon their completion of the medication administration event. As medication errors are commonly generated through a range of dynamic procedural, clinical decision-making, and human and environmental factors [28], the game was designed to be as expansive as possible for students to generate eMAR-enabled errors of all complexions, including incorrect medication dosage interpretation, administration timing, clinical indication and appropriateness, and other best practice processes deviations (eg, failure to wash hands before administration, failing to barcode scan the patient identification band or medication barcode, and failing to complete a vital signs assessment before administering an antihypertensive medication). If a student failed to attend to a specific medication administration best practice and further dependent on the severity of the error, feedback would be provided to the student on completion of the scenario, informing them of the number of errors made and brief rationale related to the mechanism of the error(s) themselves. Figure 1 provides a screenshot of an element of the game user interface, along with various manipulable objects that can spawn specific information (ie, drop-down menu to select a specific patient medication error, vital signs machine, patient dialogue, handwashing, scanning of barcodes, etc) related to the patient scenario and the medication administration context.

### Study Design

The primary study design (phase 1) will consist of a prospective, 2-armed pragmatic randomized controlled trial. Phase 2 will consist of qualitative focus group interviews, conducted after the completion of phase 1.

### Setting and Recruitment of Participants

Two waves of second-year baccalaureate nursing student participants will be contacted and recruited for participation in this proposed study at the beginning of 2 separate academic semesters: wave 1 will commence in September 2017 (fall semester) and wave 2 in January 2018 (winter semester). Due to the size of the second-year nursing student cohort (N=150) and resource limitations of the CSL, only half (n=75) of the students will partake in simulated clinical education in the CSL, per semester (split between fall and winter). Inclusion criteria for this study will be (1) second-year nursing students and (2) partaking in clinical simulated education either in fall 2017 or winter 2018 semesters. Students recruited in this study will not have had previous exposure to medication administration, and this simulated clinical experience will be their first formative interaction with medication administration and the SMART eMAR.

Before the start of each term, a researcher-led presentation will be delivered outlining the proposed study purpose, methods, and study benefits. Given the typical student enrollment within second year of the BScN program, it is expected that approximately 150 students will fit this inclusion criterion and be eligible for recruitment into the study. Participants will be offered a Can $5 gift card for a coffee shop as a nominal incentive to participate in the study. Due to the high recruitment success rate experienced in the corresponding pilot study completed in April 2016 (25/27, 93% of students sampled) [11], we estimate 120 participants will be recruited. By including at least 120 participants, phase 1 of our study will be able to detect an intervention effect size of 0.25, with a power of 0.8 and a .05 level of significance, based on a 2-tailed *t* test analysis, as calculated by G*Power 3.1 (Heinrich-Heine-Universität Düsseldorf, Germany).

### Data Collection

#### Phase 1

After recruitment and enrollment into the study (week 1), nursing students in each semester will be randomized into either the intervention or control group and will be assigned a unique participant code number. The allocation ratio will be 1:1 (intervention or control group), following a simple randomization procedure (Microsoft Excel [Microsoft Corp.] random number table). Research team members involved in data collection will be blinded to the allocation; participants will become aware of their randomization on receipt of an email from the researchers outlining their allocation into the intervention or control group. At this time, students in the intervention group will receive access to the eMAR simulation game approximately 2 weeks in advance of their in-person simulation sessions with the eMAR technology in the CSL. Along with access to the eMAR simulation game, students in the intervention group will also have access to other usual preparatory educational resources related to medication administration, such as a video demonstrating proper administration technique and a PowerPoint presentation on eMAR medication administration. To ensure a consistent duration of game intervention, students will be requested to use the eMAR simulation game for a minimum of 60 min (with no maximum) [29] before conducting their return demonstration of a medication administration using the actual SMART eMAR system in the CSL (starting week 5); analytics software operating in the background of the game will be used to track the average game play duration of participants. Students allocated to the control group will receive the usual preparatory resources related to eMAR medication administration, without access to the eMAR simulation game. At the conclusion of the trial in late October and February (week 9), all participants in the control group will receive access to the eMAR simulation game.

The duration of the active trial in each academic semester will be approximately 4 weeks, running the months of October and February. The simulated return-demonstration patient scenario that all students will be required to complete involves conducting a baseline physical assessment on a high-fidelity human mannequin simulator, performing the ordered medication administration using the SMART eMAR system, and conducting a patient follow-up assessment and documentation of the encounter in the patient’s clinical record. All participants in the intervention and control groups will receive the same patient and medication administration scenario to maximize contextual internal validity.

**Figure 2:**
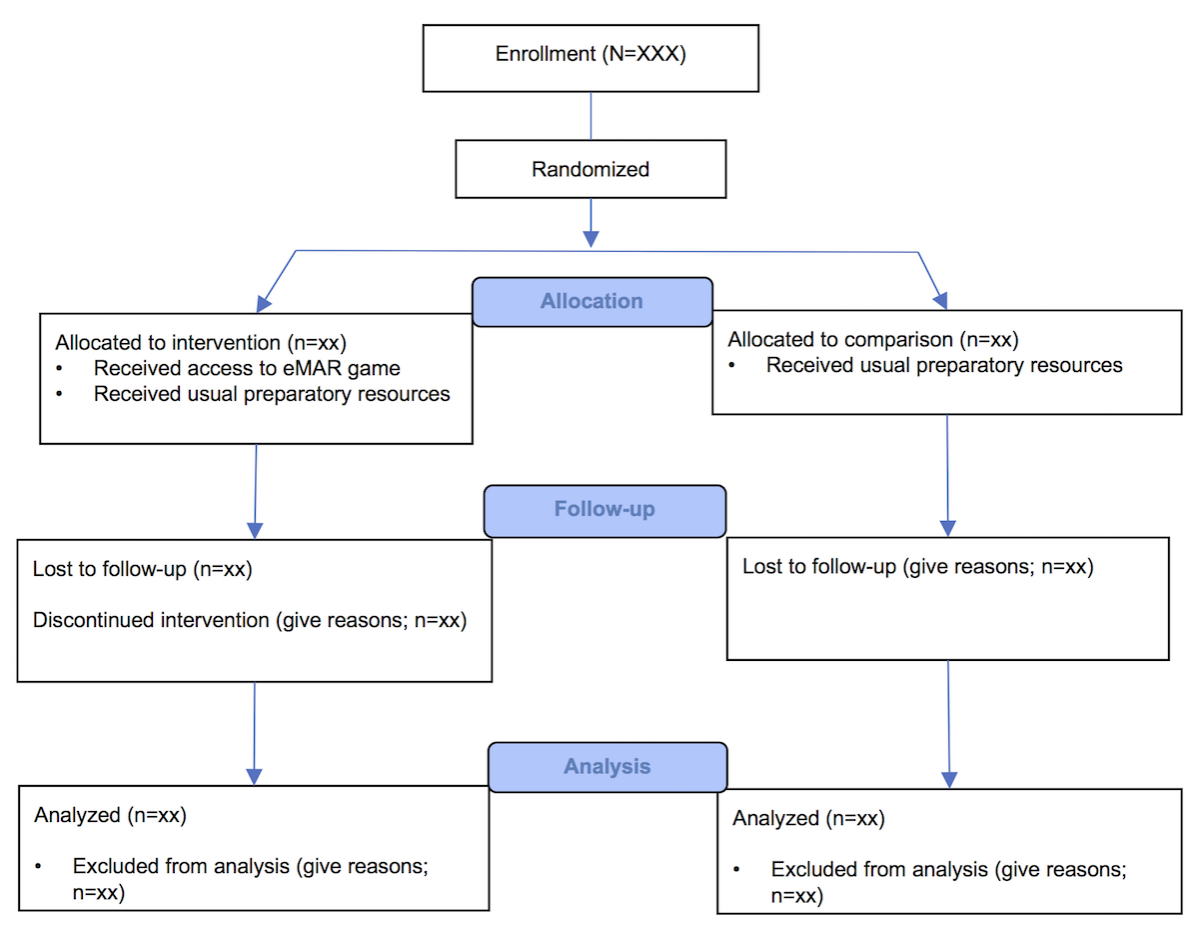
CONSORT (Consolidated Standards of Reporting Trials) diagram for electronic medication administration record (eMAR) game pragmatic trial.

Prepost data related to medication self-efficacy and knowledge will be collected through a Qualtrics (Qualtrics Inc.) Web-based survey platform before (week 4) and after the simulated return-demonstration patient scenario (week 8). Cross-sectional survey data will be collected from the intervention group only, immediately after the simulated return-demonstration patient scenario (week 8; Figure 2).

#### Outcomes

Data will be collected by 4 data collectors (nursing graduate students with clinical backgrounds using eMAR technology) who will observe nursing student participants during their return demonstration of medication administration using the SMART eMAR (weeks 5-8). These data collectors will be trained by members of the research team before the study initiation to ensure the appropriate and consistent use of the checklist to collect participant data. Mock student return demonstrations and related data collection will be conducted to improve interrater reliability; Fleiss kappa coefficients will be calculated and used to further refine interrater reliability of the data collection procedure. A formalized checklist that has been developed and used previously in an adapted form by the research team [20] will be implemented as the primary data collection instrument for phase 1. This data collection checklist will capture the following quantitative data during each participant’s medication administration return demonstration using the SMART eMAR: (1) frequency of deviations from medication administration best practices, (2) number of actual medication errors or near misses generated by the student that violate the various medication best practices and provincial regulatory requirements related to medication administration [30,31], and (3) overall duration of time required by the student to complete the entire medication administration simulated scenario. Secondary outcomes using prepost survey data related to medication self-efficacy and knowledge will be collected using questions modified from previously developed and tested instruments [32,33]. Secondary outcome cross-sectional data will also be collected using questions modified from the Huang et al’s [34] validated instrument that measures motivational (ie, attention, relevance, confidence, satisfaction) and cognitive processing of learning in game-based environments (20 questions, Cronbach alpha=.91). Measurement of motivational and cognitive processes of learning will identify levels of participant motivation related to cognitive load requirements demanded by the eMAR simulation game.

#### Phase 2

The specific questions to be asked during focus group interviews with participants will be informed by the preliminary quantitative findings [35]. Although emergent and contingent on the quantitative findings, it is expected that a range of qualitative interview questions will be developed based on the core variables in part 1 of the study (ie, medication errors generated, self-efficacy and knowledge related to administration, etc). During these focus group interviews, participant feedback related to the usability, design, and user experience of the game will also be sought. Approximately 20 participants from either semester (both intervention and control groups) will be interviewed in the qualitative phase of this study (week 9).

### Data Analysis

#### Phase 1

Survey data will be analyzed through descriptive and inferential statistics. Student *t* test to compare pre- versus postcontinuous outcomes for intervention and control groups in relation to number of medication errors or near misses, number of deviation from best practices, and length of time (efficiency) of administration will be completed.

#### Phase 2

Semistructured focus group interviews will be audio recorded and transcribed verbatim. A minimum of 2 researchers will conduct a directed content analysis [36] of the data; this will be triangulated with quantitative findings [37] to generate a deeper understanding of the effectiveness of the gamified eMAR simulation as a technology-enabled clinical education tool.

### Ethics

Ethical approval for this study has been obtained from Western University, London, Canada (HSREB #109180).

## Results

Preliminary results of half of the desired study cohort is expected in early 2018. By early spring 2018, the findings of the study in its entirety will be available. Currently, data collection of the fall 2017 cohort has concluded.

## Discussion

### Principal Considerations

Due to the pragmatic randomized controlled trial design, the findings of this study will establish baselines regarding the effects of serious gaming approaches toward improving nursing students’ eMAR medication administration safety; similarly, the findings will also outline best practices toward the development of immersive gamified simulation opportunities that can be leveraged by other educational organizations. Given the lack of research and consolidated knowledge about this teaching-learning approach [38], both quantitative and qualitative findings arising from this study will provide important theoretical and pragmatic insights to others developing immersive simulation or virtual reality opportunities.

Although still an emerging area of research, there is growing interest toward the development of serious gaming or gamified approaches in clinical education [6,39,40]. Recent evidence suggests that gamified approaches in nursing education can offer a range of benefits, including anxiety reduction in students, improvement in the repeatability of skills, and increased access to learning opportunities [41,42]. Other nursing education research has posited that there is no difference between gamified learning versus face-to-face clinical simulation [43]. Medical education research has found largely positive results regarding the use of gamification as a teaching-learning mechanism, in terms of medical content validity [27], acceptance and retention of learned knowledge [44], and ongoing skill retention and learner engagement [29,45]. The growing importance and interest in this topic can also be inferred from the recent registration of a *Cochrane Database of Systematic Review* protocol, which plans to examine the effectiveness of serious gaming and other gamified interventions in health professional education [38]. As outlined by Cant and Cooper [41], this type of clinical education pedagogy will likely “have a major place in nursing curricula in the next decade,” and therefore warrants further examination by clinician educators.

### Strengths and Limitations

As a strength, this study will be one of the first appropriately powered trials of a serious gaming intervention in nursing education. Furthermore, the pragmatic randomized controlled trial design [46] with complementing qualitative elements will assist in generating informative insights related to the effectiveness of using a serious gaming intervention to educate students to the complexities of medication administration in an eMAR environment. Although this study has some strengths, there are some limitations that should be outlined. First, the observational method and environment for data collection raise the potential of an observer effect, in that participants may recognize they are being observed by members of the research team during their return demonstration of a medication administration scenario in the CSL. Second, the variability in data collection by multiple observers may be a potential limitation. Although standardized observation guides and training will be provided to the data collectors, interrater reliability between observers may be a source of study error. Finally, given the structure of the game, it is not possible to fully ascertain whether there is a dose-response relationship between cumulative gameplay time and its effect on medication error frequency rates. As the eMAR simulation game offers the player a fair amount of autonomy to make errors and experiment with different actions and processes, any linear relationship examined between overall gameplay time of a participant and their medication error rate may not be an accurate depiction of medication administration knowledge or comprehension.

### Conclusions

Given the potential severity of health outcomes caused by medication errors, nursing education continues to place emphasis on simulated clinical education to provide students with the necessary clinical skills related to medication administration before direct interaction with real patients. Simulation has been widely adopted within clinical education to support medication administration education. However, because of financial and resource constraints experienced by educational institutions, opportunities for students to meaningfully practice eMAR administration continue to be limited. The development of innovative clinical learning strategies such as serious gaming and gamification may offer new opportunities for students to learn these important skills and develop clinical judgment. The findings of this study will generate evidence of a gamified approach to learning in nursing education and provide new pedagogical implications to prepare students with the appropriate skills, knowledge, and competencies related to electronic medication administration.

## Appendix

Multimedia Appendix 1 Funder feedback.

## Abbreviations

CSL: clinical simulation laboratory
eMAR: electronic medication administration record
SMART eMAR: a physical, simulated eMAR system used within the university’s CSL
