# Supplementary material for: Evaluating a Serious Gaming Electronic Medication Administration Record System Among Nursing Students: Protocol for a Pragmatic Randomized Controlled Trial
Source: JMIR Res Protoc. 2018 May 28;7(5):e138. doi: 10.2196/resprot.9601 (PMC5996180; doi:10.2196/resprot.9601)
Supplement: Multimedia Appendix 1 [file resprot_v7i5e138_app1.pdf]

April 5, 2017

Richard Booth
Assistant Professor
Western University

Dear Richard,

eCampusOntario is pleased to provide you with the evaluation feedback from your institution's recent submission: *Evaluation of a technology-enabled, gamified electronic medication administration record (eMAR) system for use in the simulated clinical education.*

eCampusOntario would like to thank you again for your participation in the 2016–18 Request for Proposals. Innovation in online education is another step in the journey to accessible education for all Ontarians, and we look forward to continuing this journey with you in future collaborative endeavours.

For more information please contact Jim Quick at jim@ecampusontario.ca.

Sincerely,

David Porter
CEO, eCampusOntario

<u>Proposal Evaluation Feedback</u>

Your proposal was reviewed by two peer evaluators from fellow Ontario post-secondary institutions. The following comments are from these reviewers:

<u>Evaluator 1 Comments</u>

- *Unfortunately, this evaluator did not include comments.*

<u>Evaluator 2 Comments</u>

- *This is a great proposal with an impressive team of experts. The results of this study will be of high value to universities with nursing and medical schools, but not to everyone in Ontario.*

<END OF COMMENTS>

<u>Evaluation Score</u>

The normalized score range for funded projects: **80.32–114.73**

Your normalized score: **93.83**
